# Supplementary material for: Integrin α7 is a functional cancer stem cell surface marker in oesophageal squamous cell carcinoma
Source: Nat Commun. 2016 Dec 7;7:13568. doi: 10.1038/ncomms13568 (PMC5150658; doi:10.1038/ncomms13568)
Supplement: Supplementary Information — Supplementary Figures 1-6 and Supplementary Tables 1-7 and Supplementary Methods [file ncomms13568-s1.pdf]

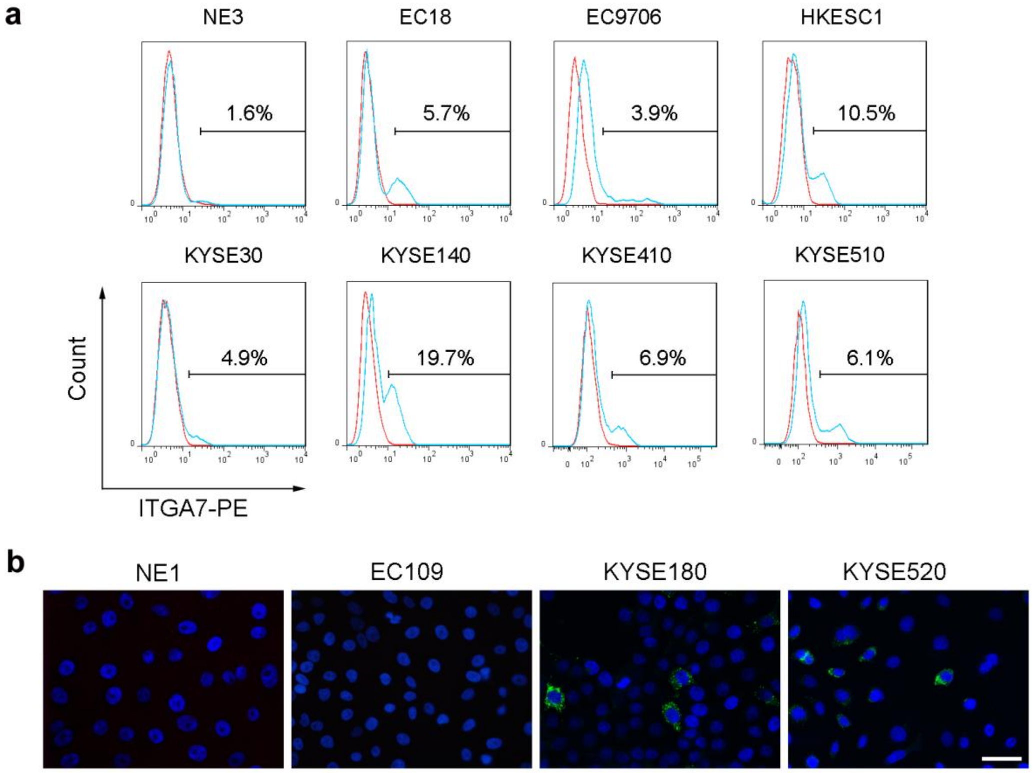

Supplementary Figure 1. ITGA7 is sporadically expressed in ESCC. (**a**) Percentage of ITGA7^{+} cells was detected by FACS in immortalized esophageal epithelial cell line NE3 and ESCC cell lines (Red: isotype control; blue: ITGA7-PE). (**b**) Representative immunofluorescence images of ITGA7^{+} cells in esophageal cell lines (green: ITGA7; blue: nuclear DAPI staining; scale bar = 50 μm).

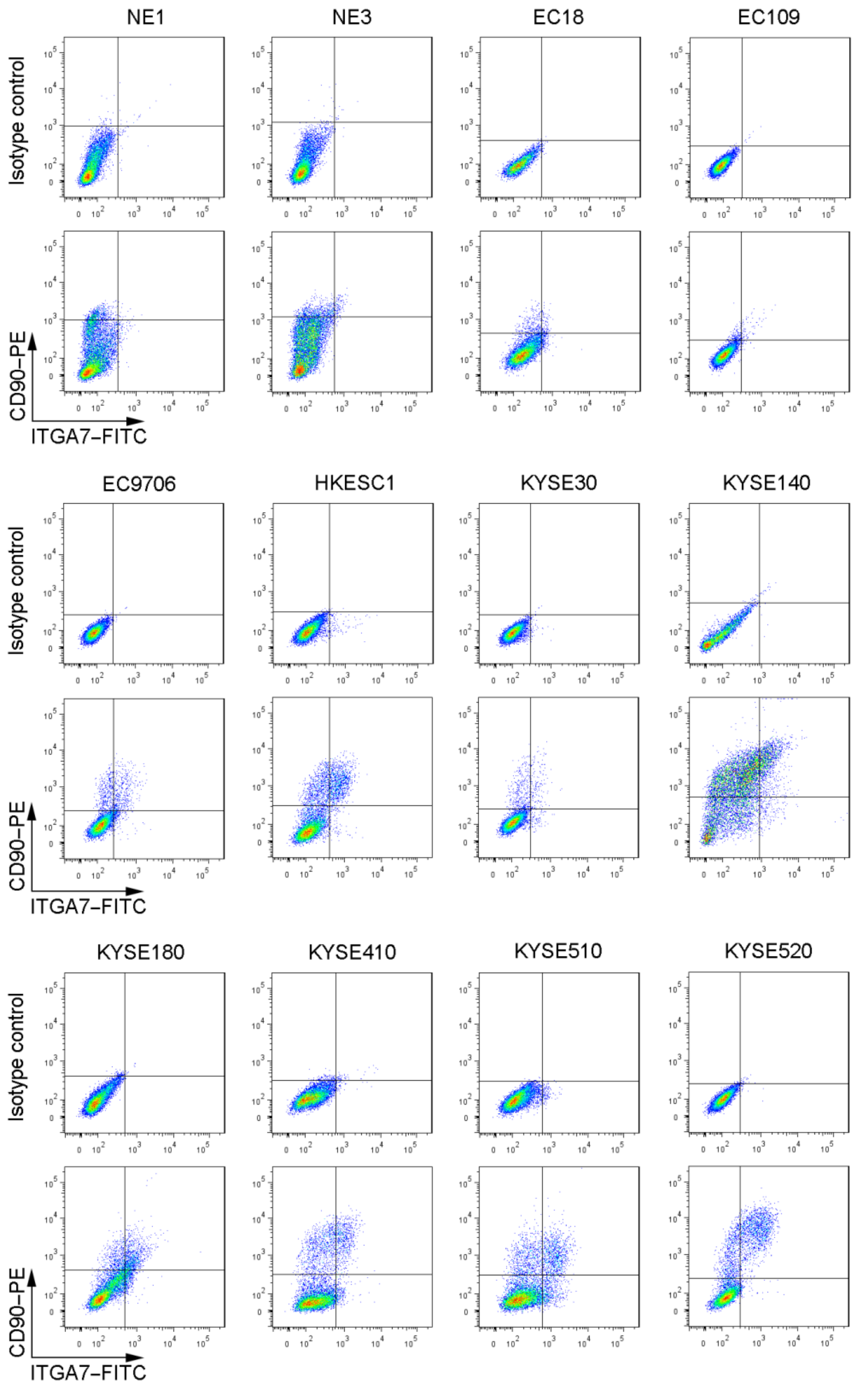

Supplementary Figure 2. The overlap expression of ITGA7 and CD90 in immortalized esophageal epithelial cell lines (NE1 and NE3) and ESCC cell lines.

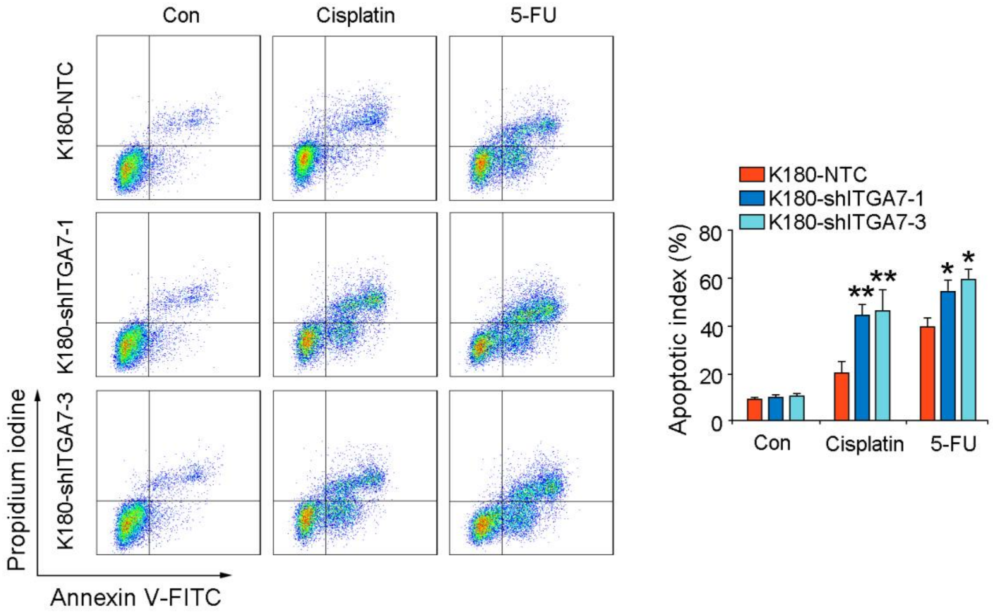

Supplementary Figure 3. Knockdown of *ITGA7* promotes apoptosis in ESCC cells. Representative of apoptosis analysis showing that sh*ITGA7*-transfected KYSE180 cells were less resistant to cisplatin and 5-FU. The apoptotic index was summarized in the bar chart and the value indicated the mean ± s.d. of three independent experiments. Statistics: ANOVA with post hoc test. *, $P < 0.05$; **, $P < 0.001$.

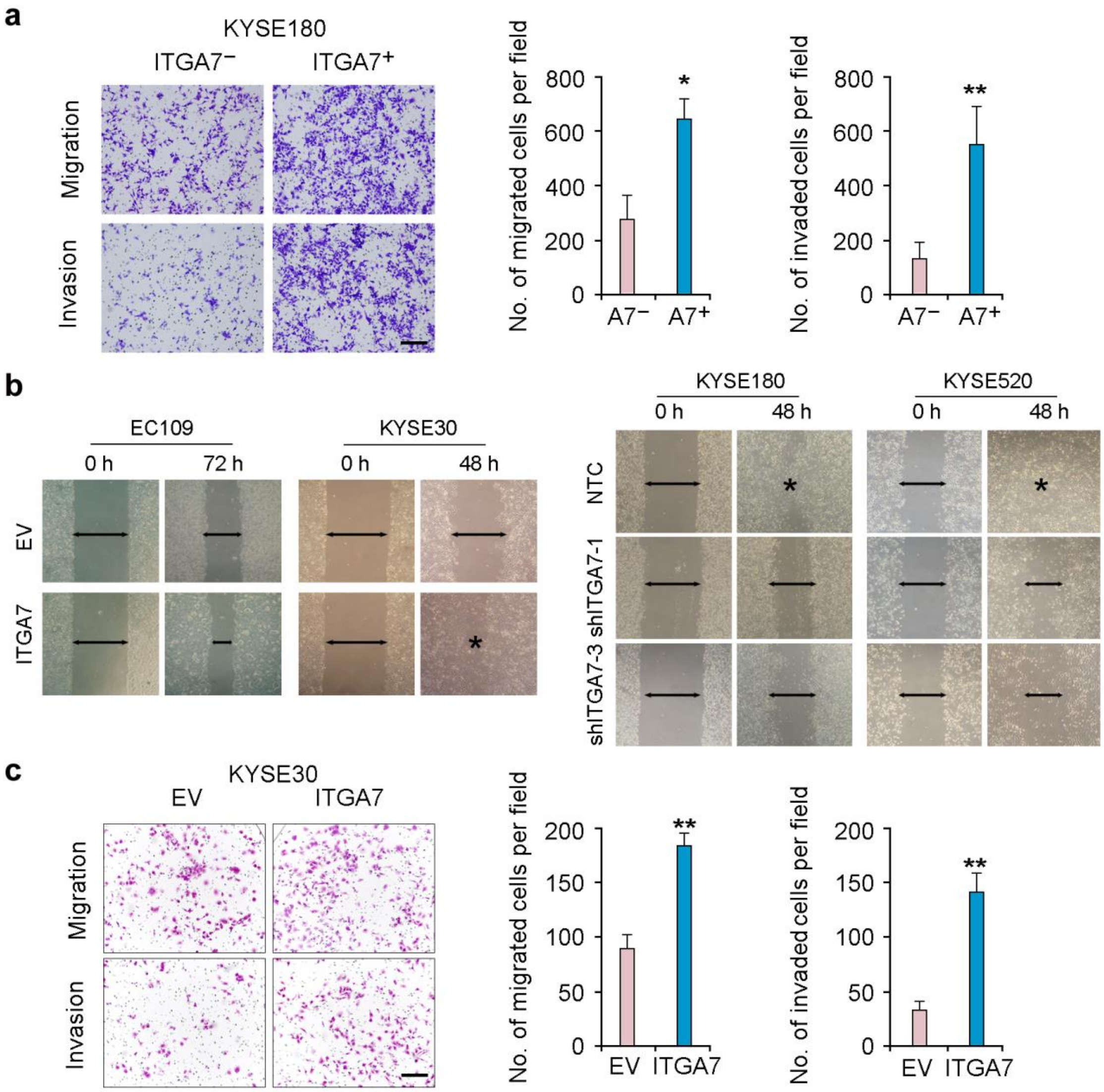

Supplementary Figure 4. ITGA7 promotes ESCC cell migration and invasion. (**a** & **c**) Representatives and summary of migration and invasion assays showing that ITGA7^{+} KYSE180 cells and *ITGA7*-KYSE30 cells exhibited enhanced migration and invasion abilities, compare to their negative counterparts. The value expressed as the mean ± s.d. of three independent experiments. Scale bar = 200 μm. (**b**) *ITGA7*-transfected cells showed higher motility in wound-healing assay, while knockdown of *ITGA7* suppressed cell motility. Statistics: (a and c) Student *t* test. *, *P*< 0.05; **, *P*< 0.001.

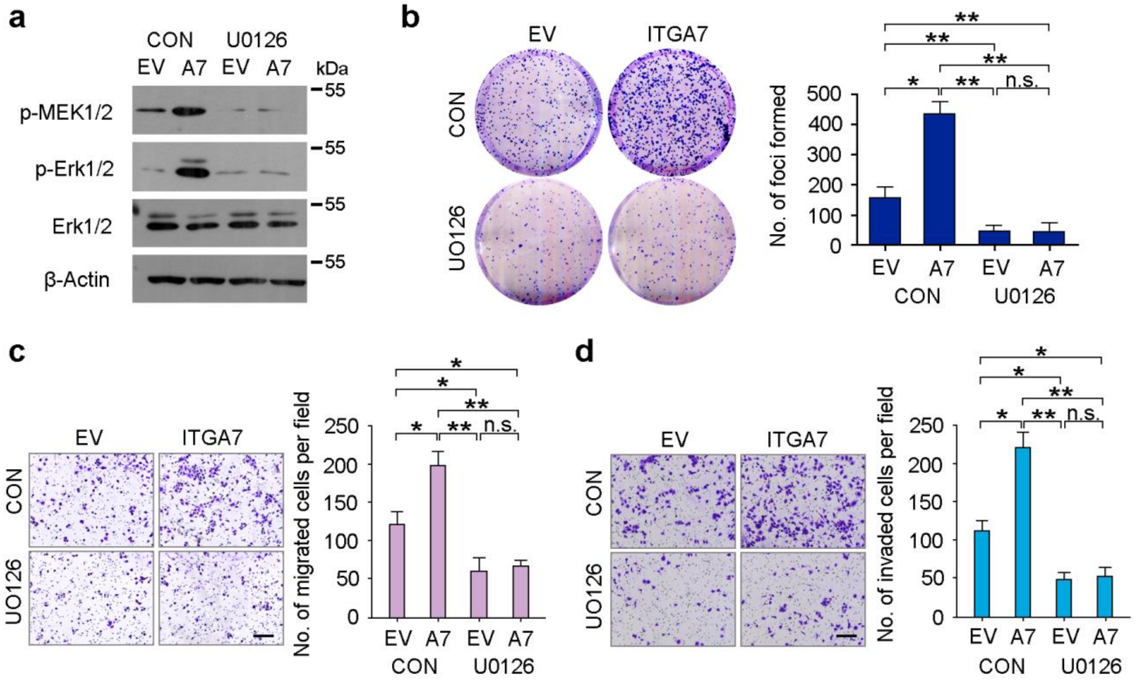

Supplementary Figure 5. MEK1/2 inhibitor suppresses ITGA7 mediated tumorigenicity and metastasis. (**a**) Western blot analyses showed that the phosphorylation of MEK1/2 and downstream player Erk1/2 was significantly blocked in *ITGA7*-transfected KYSE30 cells after treatment with 20 µM MEK1/2 inhibitor U0126 for 24 hours. Functional assays demonstrated that U0126 treatment could effectively inhibit the abilities of foci formation (**b**), cell migration (**c**; 200 µm scale bar), and invasion (**d**; 200 µm scale bar). The number of foci, migrated and invaded cells was calculated from three independent experiments and depicted as the mean ± s.d. in the bar chart. Statistics: (b, c and d) ANOVA with post hoc test. *, $P< 0.05$; **, $P< 0.001$; n.s., $P\geq 0.05$.

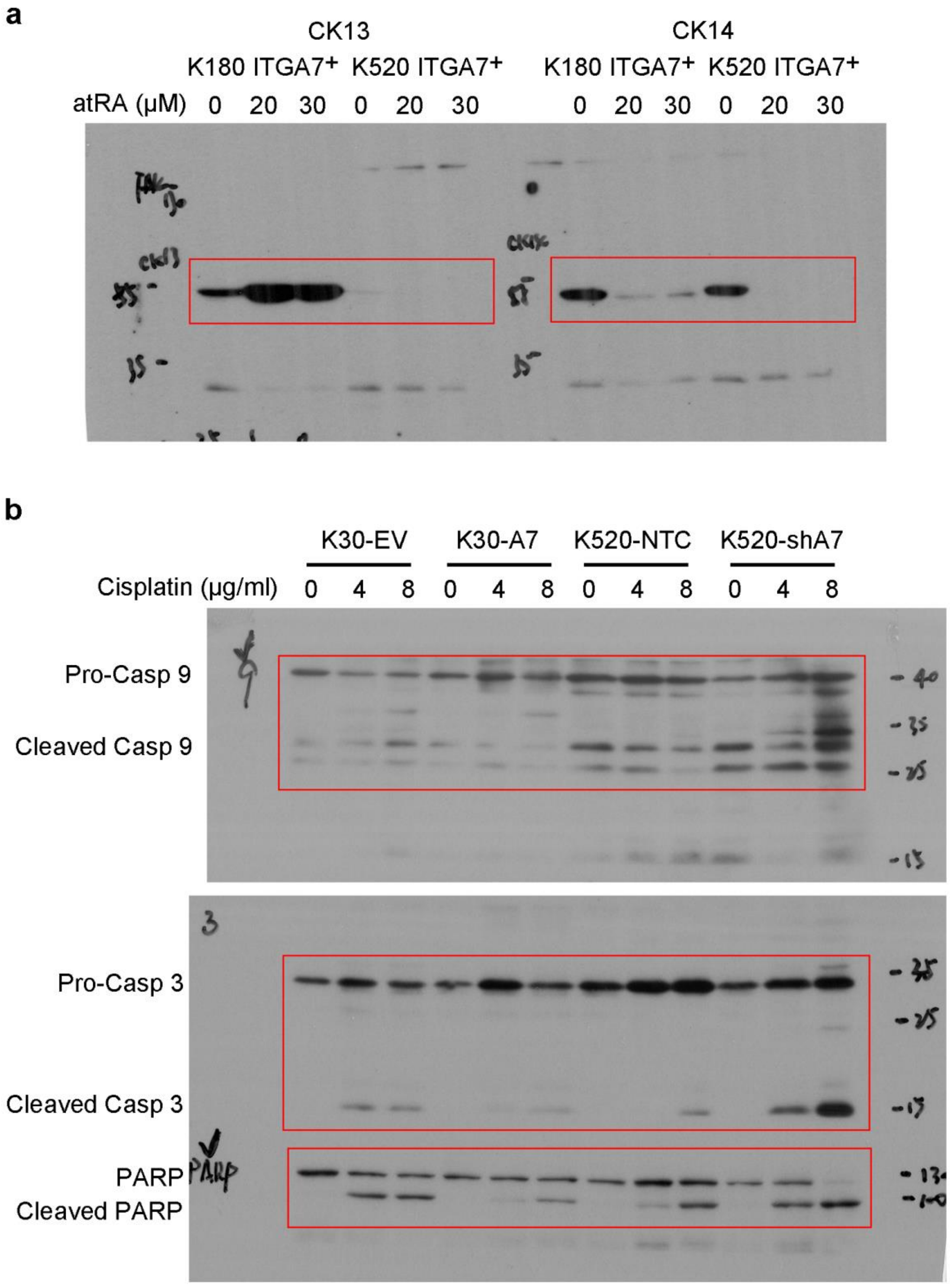

Supplementary Figure 6. (**a-b**) Full blots of Fig. 3c and Fig. 7f respectively.

Supplementary Table 1. Frequencies of $ITGA7^{+}$ cells in ESCC tumor tissues

ITGA7(%)	No. of ESCC cases
0-0.3	41
0.3-0.6	84
0.6-1	78
1-2	32
2-3	14
3-4	10
4-5	3
**Total**	**262**

Supplementary Table 2. Frequencies of $ITGA7^{+}$ and $CD90^{+}$ cells in esophageal cell lines

Cell line	$ITGA7^{+}/CD90^{+}$	$ITGA7^{+}/CD90^{-}$	$ITGA7^{-}/CD90^{+}$	$ITGA7^{-}/CD90^{-}$
NE1	0.29%	1.33%	7.04%	91.34%
NE3	1.20%	0.21%	7.30%	91.29%
EC18	2.95%	1.98%	6.47%	88.60%
EC109	0.52%	0.81%	0.97%	97.70%
EC9706	2.20%	1.53%	3.08%	93.19%
HKESC1	11.10%	1.03%	7.74%	80.13%
KYSE30	3.80%	1.02%	4.88%	90.30%
KYSE140	16.80%	1.30%	50.80%	31.10%
KYSE180	13.50%	4.26%	7.40%	74.84%
KYSE410	6.22%	2.32%	8.21%	83.25%
KYSE510	5.50%	1.40%	11.50%	81.60%
KYSE520	17.10%	1.48%	7.52%	73.90%

Supplementary Table 3. Overlap expression of ITGA7 and CD90

Cell line	$ITGA7^{+a}$	$CD90^{+b}$	$ITGA7^+/CD90^+$ in total $ITGA7^{+c}$
NE1	1.62%	7.33%	17.90%
NE3	1.41%	8.50%	85.11%
EC18	4.93%	9.42%	59.84%
EC109	1.33%	1.49%	39.10%
EC9706	3.73%	5.28%	58.98%
HKESC1	12.13%	18.84%	91.51%
KYSE30	4.82%	8.68%	78.84%
KYSE140	18.10%	67.60%	92.82%
KYSE180	17.76%	20.90%	76.01%
KYSE410	8.54%	14.43%	72.83%
KYSE510	6.90%	17.00%	79.71%
KYSE520	18.58%	24.62%	92.03%

[a]Calculation formula: $CD90^+/ITGA7^+\% + CD90^-/ITGA7^+\%$;
[b]Calculation formula: $CD90^+/ITGA7^+\% + CD90^+/ITGA7^-\%$;
[c]Calculation formula: $CD90^+/ITGA7^+\% / (CD90^+/ITGA7^+\% + CD90^-/ITGA7^+\%)$.

Supplementary Table 4. List of antibodies used in this project

Antibody	Cat No.	Vendor	Application
Mouse anti-human ITGA7	AT2566a	Abgent	WB, 1:1000; IHC FACS IF, 1:100
Rabbit anti-human ITGA7	AP18839a	Abgent	FACS IF, 1:100
PE mouse anti-human CD90	555596	BD Pharmingen	FACS, 1:20
Purified mouse anti-human CD90	555593	BD Pharmingen	IF, 1:50
Mouse anti-human CD90	ab225	Abcam	WB, 1:500
Rabbit anti-human FAK	#13009	Cell Signaling	WB, 1:1000
Rabbit anti-human FAK (Tyr397)	#8556	Cell Signaling	WB, 1:1000
Rabbit anti-human Src	#2109	Cell Signaling	WB, 1:1000
Rabbit anti-human Src (Tyr416)	#6943	Cell Signaling	WB, 1:1000
Rabbit anti-human c-Raf	#9422	Cell Signaling	WB, 1:1000
Rabbit anti-human c-Raf (Ser259)	#9421	Cell Signaling	WB, 1:1000
Rabbit anti-human MEK1/2 (Ser217/221)	#9154	Cell Signaling	WB, 1:1000
Rabbit anti-human Erk1/2	#9102	Cell Signaling	WB, 1:1000
Rabbit anti-human Erk1/2 (Thr202/204)	#9101	Cell Signaling	WB, 1:1000
Mouse anti-human β-actin	ab6276	Abcam	WB, 1:5000
Rabbit anti-human E-Cadherin	#3195P	Cell Signaling	WB, 1:1000
Mouse anti-human β-Catenin	#9562	Cell Signaling	WB, 1:1000
Mouse anti-human Fibronectin	ab6328	Abcam	WB, 1:500
Mouse anti-human Vimentin	#3390S	Cell Signaling	WB, 1:1000
Mouse anti-human α-SMA	A2547	Sigma-Aldrich	WB, 1:2000
Rabbit anti-human Akt	#9272	Cell Signaling	WB, 1:1000
Rabbit anti-human Akt (Ser473)	#9271	Cell Signaling	WB, 1:1000
Rabbit anti-human Caspase-9	#9502	Cell Signaling	WB, 1:1000
Rabbit anti-human Caspase-3	#9662	Cell Signaling	WB, 1:1000
Rabbit anti-human PARP	#9542	Cell Signaling	WB, 1:1000
Rabbit anti-human CK13	NBP2-16086	Novus biological	WB, 1:2000
Rabbit anti-human CK14	NBP1-84917	Novus biological	WB, 1:2000
Rabbit anti-human OCT3/4	#2750	Cell Signaling	WB, 1:4000
Rabbit anti-human SOX2	#3579	Cell Signaling	WB, 1:4000
Rabbit anti-human NANOG	#4903	Cell Signaling	WB, 1:4000
Goat anti-mouse IgG HRP	A4416	Sigma-Aldrich	WB, 1:10000
Goat anti-rabbit IgG HRP	A9169	Sigma-Aldrich	WB, 1:10000
Mouse IgG2a Isotype Control	M076-3	MBL	FACS, 1:200
Rabbit IgG Isotype Control	SC-3888	Santa Cruz	FACS IF, 1:100
PE mouse IgG1 Control	12-4714-82	eBioscience	FACS, 1:20
PE anti-mouse IgG2a	SC-3765	Santa Cruz	FACS, 1:100
CF^{TM}488 goat anti-mouse IgG	20014-1	Biotium	FACS IF, 1:5000
CF^{TM}488 goat anti-rabbit IgG	20012-1	Biotium	FACS IF, 1:5000

Supplementary Table 5. List of PCR primers for expression, cloning and BGS

Gene Name	Primer Sequence (5'-3')
**For Real-time PCR**	
ITGA7<br>NM_002206.2	Forward: CTGACTCCATGTTCGGGATCA<br>Reverse: CACCTGTGAAGGTTTGGCG
OCT3/4<br>NM_002701	Forward: CTTGCTGCAGAAGTGGGTGGAGGAA<br>Reverse: CTGCAGTGTGGGTTTCGGGCA
SOX2<br>NM_003106	Forward: AAATGGGAGGGGTGCAAAAGAGGAG<br>Reverse: CAGCTGTCATTTGCTGTGGGTGATG
NANOG<br>NM_024865	Forward: AATACCTCAGCCTCCAGCAGATG<br>Reverse: TGCGTCACACCATTGCTATTCTTC
NOTCH1<br>NM_017617	Forward: CCTGAGGGCTTCAAAGTGTC<br>Reverse: CGGAACTTCTTGGTCTCCAG
GAPDH<br>NM_001256799	Forward: GGAGCGAGATCCCTCCAAAAT<br>Reverse: GGCTGTTGTCATACTTCTCATGG
**For full length cDNA**	
ITGA7<br>NM_002206.2	Forward: *gctctaga*GATTTCCCTTGCATTCGCTGGGAGCTC<br>Reverse: *cgggatcc*CTAGGCGGTGCCTGGCCCTGGAT
**For BGS**	
ITGA7-NCG1<br>+19,539 - +19,955	Forward: AGGAGGTAAGGGGAAGGGAGGG<br>Reverse: CCCACCTCCAAACCTCACCTTC
ITGA7-NCG2<br>+13,679 - +14,162	Forward: ATTGGAGGGAGGGGGTATTGAT<br>Reverse: TCCCCCAACTAAAACCCACAAC
ITGA7-NCG3<br>+4,167 - +4,600	Forward: GTTTTTGTTGGGTTTTTGATGA<br>Reverse: AATATCAATTTTCTCAAACCCT

Supplementary Table 6. List of reagents used in this project

reagents	Cat No.	Vendor
5-aza-2'-deoxycytidine (5-aza-dC)	A3656	Sigma-Aldrich
All-trans retinoic acid (atRA)	R2625	Sigma-Aldrich
1,2,4,5-Benzenetetraamine tetrahydrochloride (Y15)	305065	Sigma-Aldrich
U0126	#9903	Cell Signaling

Supplementary Table 7. The shRNA sequences for lentiviral transduction

Gene	shRNA Sequence
ITGA7 (NM_002206.2)	TRCN0000057708:CCGGCCCAGGAACCTATAATTGGAACTCG AGTTCCAATTATAGGTTCCTGGGTTTTTG
	TRCN0000057711:CCGGGTCCTCCATAAAGAACTTGATCTCG AGATCAAGTTCTTTATGGAGGACTTTTTG
Non-Target Control	SHC002:CCGGCAACAAGATGAAGAGCACAACTCGAGTTGGT GCTCTTCATCTTGTTGTTTTT

SUPPLEMENTARY METHODS

Wound healing assay. Cell mobility was studied by a scratch wound-healing assay. Briefly, cells were cultured in a 6-well plate until confluence. The culture medium was replaced by serum-free medium 24 h before wound creation. The cell layer was wounded using a sterile tip. After wounding, the medium was changed to fresh serum-free medium to remove cellular debris. Serial photographs were obtained at different time points.

Xenograft processing and cell dissociation. Xenografts dissected from mice were dissociated into single cells using Liberase TM (Roche) which contained a blend of collagenase I and collagenase II. Before processing, tumor tissues were rinsed with PBS once and then rinsed again with DMEM/F-12 (Gibco) supplemented with 1% penicillin/streptomycin (P/S). For every 1 cm^3 tissue, 5 μg per ml Liberase enzymes were added to DMEM/F-12 medium for dissociation. Tumor tissues were minced into less than 1 mm^3 pieces in the presence of supplemented medium in a cell culture dish under sterile condition. The cell suspension was subsequently filtered through a 100 μm cell strainer and washed with DMEM/F-12 medium. The cells were cultured in complete DMEM medium supplemented with 10% FBS and 1% P/S.
